# IL-6 Production by Dendritic Cells Is Dispensable for CD8^+^ Memory T-Cell Generation

**DOI:** 10.1155/2013/126189

**Published:** 2012-12-30

**Authors:** Jean-François Daudelin, Mélissa Mathieu, Salix Boulet, Nathalie Labrecque

**Affiliations:** ^1^Maisonneuve-Rosemont Hospital Research Center, University of Montreal, 5415 Boulevard de l'Assomption, Montréal, QC, Canada H1T 2M4; ^2^Department of Microbiology and Immunology, University of Montreal, Montréal, QC, Canada H3C 3J7; ^3^Department of Medicine, University of Montreal, Montréal, QC, Canada H3C 3J7

## Abstract

Following activation, naïve CD8^+^ T cells will differentiate into effectors that differ in their ability to survive: some will persist as memory cells while the majority will die by apoptosis. Signals given by antigen-presenting cells (APCs) at the time of priming modulate this differential outcome. We have recently shown that, in opposition to dendritic cell (DC), CD40-activated B-(CD40-B) cell vaccination fails to efficiently produce CD8^+^ memory T cells. Understanding why CD40-B-cell vaccination does not lead to the generation of functional long-lived memory cells is essential to define the signals that should be provided to naïve T cells by APCs. Here we show that CD40-B cells produce very low amount of IL-6 when compared to DCs. However, supplementation with IL-6 during CD40-B-cell vaccination did not improve memory generation. Furthermore, IL-6-deficient DCs maintained the capacity to promote the formation of functional CD8^+^ effectors and memory cells. Our results suggest that in APC vaccination models, IL-6 provided by the APCs is dispensable for proper CD8^+^ T-cell memory generation.

## 1. Introduction

The recognition of a foreign antigen (Ag) presented by specialized Ag-presenting cells (APCs) in lymphoid organs by naïve CD8^+^ T cells leads to their activation, differentiation, and proliferation. This is accompanied by changes in migration properties and gain of effector functions to control the infection. After elimination of the pathogen, most (90–95%) of the activated CD8^+^ effector T cells (Te) die during the contraction phase to reset the system for the next challenge. Importantly, a fraction of the Ag-specific Te cells will survive as resting memory T cells (Tm) able to respond quickly to a second Ag encounter.

During acute infection, two subsets of CD8^+^ effectors, short-lived effector cells (SLECs; CD127^lo^ and KLRG-1^hi^), and memory precursor effector cells (MPECs; CD127^hi^ and KLRG-1^lo^) can be identified at the peak of the response [1–6]. Only MPECs, which represent about 10% of the Ag-specific population at the peak of the response, survive and further differentiate into Tm cells [1–5]. However, a different picture emerged in vaccination strategies using Ag-pulsed APCs [2, 7–11] or Ag plus adjuvant [8, 12]. We and others have shown that CD8^+^ T-cell response to immunization with TLR-stimulated DCs follows a different course than response to infection [2, 7–10]. Due to low inflammation, the majority of CD8^+^ Te cells acquire an MPEC phenotype at the peak of the response [2, 7–10]. These MPECs are very good effectors endowed with the ability to produce cytokines and kill target cells [10, 11]. Unlike the MPECs that are generated following infection, MPECs obtained following DC vaccination will still undergo a normal contraction phase [7, 8] and thus only a fraction of them will become long-lived Tm cells. Similarly, vaccination with Ag plus adjuvant generates a high proportion of CD127^hi^ cells (MPECs) at the peak of the response and only a fraction of them will survive as long-lived CD8^+^ Tm cells [8, 12]. Following vaccination with Ag plus adjuvant, it was shown that high level of expression of IL-6 receptor (R) *α* chain in combination with high level of expression of IL-7R*α* (CD127) better identifies the MPECs that will further differentiate into Tm cells [12]. This suggests that IL-6 signal might contribute to Tm-cell development.

Until recently, little was known about the potential of other APCs, such as B cells, to induce a CD8^+^ T-cell response [11, 13–15]. We and others have shown that CD40-activated B (CD40-B) cells can prime a functional CD8^+^ T cell response *in vivo *[11, 13–15]. We have shown that as for DC vaccination, all effectors acquire a MPEC phenotype following CD40-B-cell immunization [11]. Furthermore, these MPECs have excellent effector functions as measured by their ability to secrete cytokines, kill target cells *in vivo* and clear a bacterial infection [11]. Although MPECs were generated with CD40-B-cell vaccination, Tm-cell generation was inefficient [11]. Therefore, understanding why CD40-B cell vaccination does not lead to the formation of functional long-lived Tm cells is essential to define the signals that should be provided to naïve T cells by APCs to promote efficient Tm-cell differentiation. The reported high level of expression of IL-6R*α* by prememory CD8^+^ T cells [12] suggests that IL-6 may be one of the missing signal.

 IL-6 was first identified as a B-cell proliferation and differentiation factor [16]. Its high affinity receptor is composed of the IL-6R*α* chain and the common gp30 chain [16]. As many cytokines, IL-6 has pleiotropic action on different cell types of the immune system [16]. Specifically, on CD8^+^ T cells, IL-6 was reported to promote the survival of naïve T cells [17–20], to enhance the proliferation of CD8^+^ T cells following TCR triggering [14, 20–23] and to synergize with IL-7 or IL-15 to induce Ag-independent proliferation of CD8^+^ T cells [24]. IL-6 was also shown to contribute to *in vivo* CD8^+^ T-cell response. Indeed, maximal *in vivo* CD8^+^ T cell proliferation following vaccination with CD40-B cells stimulated via the B cell receptor and TLR7 was dependent on IL-6 production by B cells [14]. Moreover, cytotoxic CD8^+^ T-cell differentiation was dependent on IL-6 induction by adjuvant in vaccination protocol [25]. Finally, the transfer of CD8^+^ MPECs into IL-6-deficient hosts severely impaired the generation of long-lived CD8^+^ Tm cells [12]. These studies suggest that IL-6 is essential for optimal and complete *in vivo* response of CD8^+^ T cells.

The reported influences of IL-6 on CD8^+^ T-cell response lead us to investigate whether IL-6 signal from APCs during priming was necessary to promote the formation of CD8^+^ Tm cells following APC vaccination. In this paper, we show that CD40-B cells stimulated with LPS produce very low amount of IL-6 when compared to DCs and that supplementation with IL-6 during CD40-B-cell vaccination did not improve their ability to generate CD8^+^ Tm cells. Furthermore, vaccination with IL-6-deficient DCs did not impede their ability to promote the formation of functional CD8^+^ effectors and memory T cells.

## 2. Materials and Methods

### 2.1. Mice

B6.SJL and OT-I [26] mice were bred at the Maisonneuve-Rosemont Hospital Research Center facility. IL-6 knock-out (KO) (B6.129S2-*Il6* ^tm1Kopf^/J) mice [27] were purchased from The Jackson Laboratory. Mice were housed in a pathogen-free environment and treated in accordance to the Canadian Council on Animal Care guidelines. Our animal protocol (number: 2007-36) was approved by the Maisonneuve-Rosemont Hospital Research Center Animal Care Committee.

### 2.2. B-Cell and DC Cultures

For B-cell culture, lymphocytes were isolated on a FICOLL gradient from male B6.SJL spleen followed by a 4 days culture on irradiated fibroblasts stably transfected with the CD40L cDNA (3T3-CD40L) to generate CD40-B cells [28]. Bone-marrow-derived DCs were generated as previously described [8]. The day before harvesting, lipopolysaccharide (LPS) (1 *μ*g/mL) was added to DC and CD40-B-cell cultures. The ovalbumin (OVA_257–264_) peptide (SIINFEKL) (Midwest biotech) was loaded overnight on DCs (2 *μ*g/mL) and B cells (4 *μ*g/mL).

### 2.3. Immunization and Analysis of T-Cell Responses

Two days after adoptive transfer of 10^6^ OT-I T cells (CD45.2^+^; from female mice) into female B6.SJL mice (CD45.1^+^), recipients were immunized intravenously (i.v.) with 0.5 × 10^6^ DCs or 2 × 10^6^ DCs (as indicated in the Figure legend) or 2 × 10^6^ CD40-B cells from male mice to induce a CD4^+^ T-cell response against the male minor histocompatibility antigen HY [29]. Some mice were injected intraperitoneally (i.p) with 500 ng of recombinant mouse IL-6 (R&D Systems). The presence of Te (d4 post-immunization) and Tm (d45 postimmunization) cells was evaluated in the same mouse by sequential removal of superficial lymph nodes as described previously [8]. Functions of Te (d4) and Tm (d60) were analyzed as previously described with minor modifications [8]. Splenocytes were restimulated with 2 *μ*g/mL OVA_257–264_ peptide in complete RPMI 1640 for 6 h at 37°C. For the last 3 h, 10 *μ*g/mL of brefeldin A (Sigma Aldrich) was added. Te and Tm cells were identified by flow cytometry as being CD8^+^ and CD45.2^+^.

### 2.4. Mouse Surgery

Lymph node removal by surgery was done as described [30]. Briefly, mice were anesthetised by inhalation of isoflurane (2%, 1L oxygen). Before the surgery, eye ointment was applied to avoid eye dryness and buprenorphine was administered subcutaneously (0.05–0.1 mg/Kg) as an analgesic. To harvest the brachial and the inguinal lymph nodes, a small incision (5 mm) of the skin was made and the lymph nodes were removed using forceps. The incision was closed with one clip (Michel suture clips, 7.5 × 1.75 mm, Harvard Apparatus).

### 2.5. Antibodies, Cytometry, and ELISA

Anti-CD86 (GL-1), -TNF-*α* (MP6-XT22), and -Bcl-2 (3F11) antibodies were purchased from BD Biosciences. Anti-H-2K^b^ (AF6-88.5), -CD45.2 (104), -CD44 (1M7), -CD8 (53-6.7), -CD19 (6D5), -CD11c (N418), -CD80 (16-10A1), -IL-6R*α* (D7715A7), -CD43 (1B11), -CD62L (MEL-14), and -IL-2 (JES6-5H4) antibodies were purchased from Biolegend. Anti-I-A^b^ (28-16-8S) was purchased from Cedarlane. Anti-CD127 (A7R34), -Eomes (Dan11mag), -KLRG1 (2F1), and -granzyme B (16G6) antibodies were purchased from eBioscience. Anti-Bcl-6 (7D1) antibody was purchased from Santa Cruz Biotechnology. Anti-CXCR3 (220803) antibody was purchased from R&D Systems. Anti-IFN-*γ* (XMG1.2) antibody was purchased from Life technologies. OVA peptide loading on K^b^ MHC was measured by staining with the 25-D1.16 Ab [31] followed by staining with a rat anti-mouse IgG1 (A85-1) antibody from BD Biosciences. Cell surface and intracellular stainings for cytokines were performed as previously described [8, 32]. Bcl-6 and Eomes intracellular stainings were performed with the FoxP3 kit from eBioscience. For Bcl-2 staining, cells were stained for 30 minutes in 0.1% saponin (Sigma-Aldrich) and washed twice without saponin before cell surface staining. All stainings were analyzed on a BD FACSCanto I system.

For ELISA, B cells and DCs were cultured as described above. Before harvesting, supernatants were collected and ELISA was performed against IL-6 (Biolegend), according to the manufacturer's protocol.

### 2.6. Statistical Analysis

Statistical analyses for differences between groups were performed using Mann Whitney test (two experimental groups) or one-way ANOVA followed by Games-Howell posttest (3 experimental groups or more). Data are presented as mean ± standard error of the mean (SEM). All tests were two-sided and *P* < 0.05 was considered statistically significant. *P < 0.05, **P < 0.01, ***P < 0.001 and NS: non-significant.

## 3. Results and Discussion

### 3.1. Expression of IL-6R*α* by CD8^+^ T Cells following Vaccination with APCs

**Figure 1:**
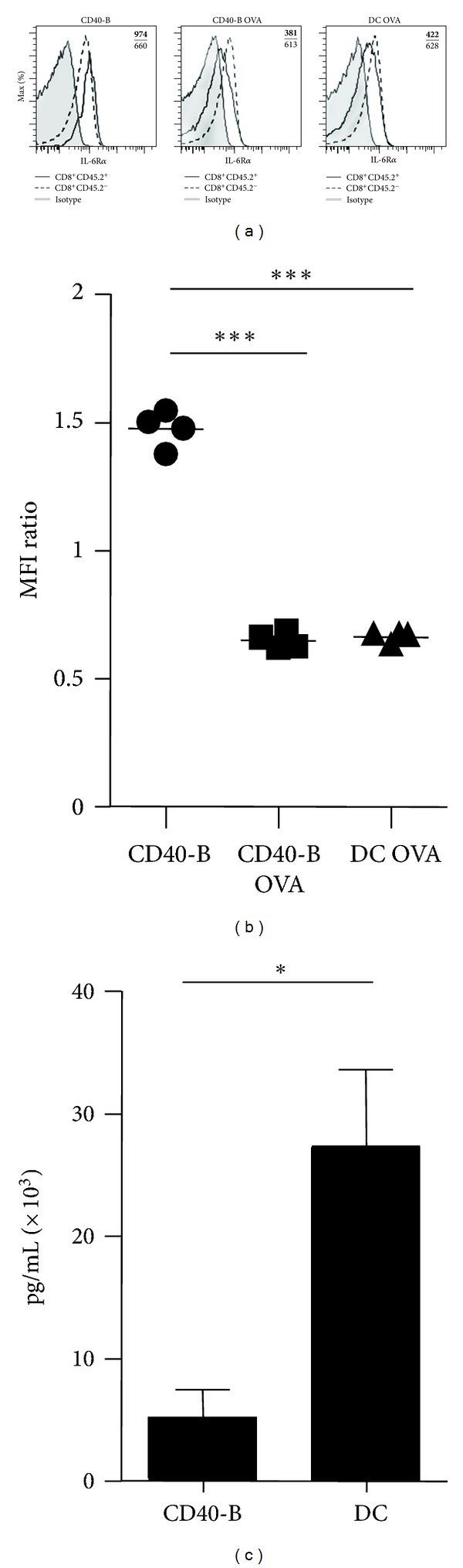
CD40-B cell and DC immunizations generate effectors expressing similar levels of IL-6R*α*. (a) Expression of IL-6R*α* by OVA-specific Te cells at the peak of the T cell response (day 4). 10^6^ female OT-I T cells (CD8^+^CD45.2^+^) were adoptively transferred into congenic B6.SJL female mice (CD45.1^+^) followed by immunization two days later with 2 × 10^6^ LPS-matured unloaded CD40-B cells (CD40-B), LPS-matured CD40-B cells loaded with the OVA peptide (CD40-B OVA) or LPS-matured DCs loaded with the OVA peptide (DC OVA). The representative overlay histograms show expression of IL-6R*α* by OVA-specific Te cells (CD8^+^CD45.2^+^) and endogenous T cells (CD8^+^CD45.2^−^). The upper bold number indicates the mean fluorescence intensity (MFI) of OVA-specific Te cells while the lower number is for the endogenous CD8^+^ T cells. (b) Quantification of IL-6R*α* expression by effectors. The MFI of IL-6R*α* expression by effector CD8^+^ T cells (CD8^+^CD45.2^+^) was normalized to the MFI of the recipient CD8^+^ T cells (CD8^+^CD45.2^−^). Each dot represents one mouse. (c) CD40-B cells produce less IL-6 than DCs. Supernatants from CD40-B LPS or DC LPS culture were used to measure IL-6 secretion by ELISA. Mean ± SEM is shown for 3 independent experiments. *P < 0.05 and ***P < 0.001.

Our previous work has shown that vaccination with CD40-B cells matured with LPS and loaded with the OVA peptide leads to the formation of functional CD8^+^ Te cells but not Tm cells [11]. Although the CD8^+^ Te cells generated following CD40-B cell vaccination were enriched for MPECs (CD127^hi^ and KLRG1^lo^), they did not survive the contraction phase [11]. Since high level of IL-6R*α* expression was shown to better identify at the peak of the T-cell response the MPECs that will differentiate into CD8^+^ Tm cells [12], we have evaluated if the MPECs generated following CD40-B-cell vaccination express high level of IL-6R*α*. As shown in Figures 1(a) and 1(b), at the peak of the T-cell response (day 4 in this model) most of the OVA-specific CD8^+^ Te cells express high level of IL-6R*α*. Furthermore, the CD8^+^ Te cells generated following CD40-B-cell vaccination express similar level of IL-6R*α* than those obtained with DC vaccination (Figures 1(a) and 1(b)), which efficiently generates CD8^+^ Tm cells. These results indicate that MPECs generated following CD40-B cell vaccination should be able to respond to IL-6 during the contraction phase of the response. The fact that CD40-B-cell vaccination generates MPECs expressing high levels of both IL-7R*α* (Supplemental Figure 1 and ref [11] see Supplementary Materials available online at doi:10.1155/2012/126189.) and IL-6R*α* suggests that these MPECs should received the proper survival signals allowing them to persist during the contraction phase and further differentiate into Tm cells. However, our previous work has shown that the MPECs obtained with CD40-B cell vaccination rapidly contract during the T cell response and do not differentiate into CD8^+^ Tm cells [11]. This suggests that other survival and differentiation factors might be implicated for the differentiation of MPECs into CD8^+^ Tm cells.

### 3.2. IL-6 Supplementation Does Not Enhance CD8^+^ Tm-Cell Generation following CD40-B-Cell Vaccination

The reported role of IL-6 in CD8^+^ T-cell proliferation and differentiation [12, 14, 20–23, 25] leads us to evaluate if CD40-B cells were providing IL-6 during the priming of naïve CD8^+^ T cells. IL-6 was quantified in the supernatants obtained at the end of CD40-B-cell and DC cultures. As shown in Figure 1(c), CD40-B cells produce around 5-fold less IL-6 than DCs. This reduced production of IL-6 might be responsible for the lack of CD8^+^ Tm-cell generation with CD40-B-cell vaccination.

**Figure 2:**
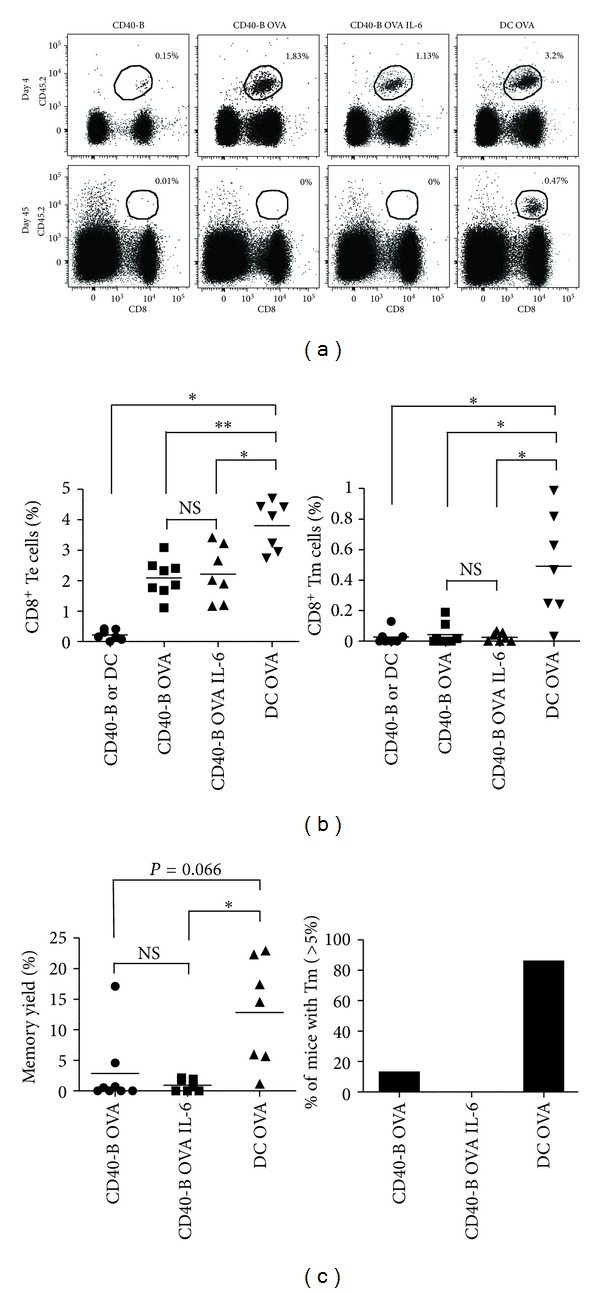
IL-6 supplementation does not increase the generation of CD8^+^ memory T cells following CD40-B cell immunization. (a) CD40-B cell vaccination with or without IL-6 co-injection generates Te cells but not Tm cells. Immunizations were performed as in Figure 1. One group of CD40-B cell vaccinated mice received recombinant IL-6 (500 ng, i.p.). OVA-specific T cells (CD8^+^CD45.2^+^) were analyzed in the same mouse by surgical removal of superficial lymph nodes at day 4 (effector) and day 45 (memory) post-immunization. The percentage of Te and Tm cells generated are indicated on each dot plot. (b) Percentage of CD8^+^ Te (day 4, left panel) and Tm (day 45, rigth panel) cells in one lymph node is shown. (c) Efficiency of CD8^+^ Tm cell generation. Left panel shows the yield of Tm cell formation calculated as the percentage of Te cells that develop into Tm cells while the right panel shows the percentage of mice that generates more than 5% of CD8^+^ Tm cells for the different immunization conditions. The results are from three independent experiments. *P < 0.05 and **P < 0.01.

To test whether the decreased IL-6 production by CD40-B cells was responsible for their inability to induce CD8^+^ Tm-cell development, we injected IL-6 at the time of CD40-B-cell immunization. The dose of IL-6 was chosen based on previous publications where IL-6 injection had an effect on T-cell response [33, 34]. As shown in Figure 2, the administration of IL-6 (500 ng) i.p. at the time of OT-I naïve CD8^+^ T-cell priming by CD40-B cells did not enhance the generation of CD8^+^ Te and Tm cells. Furthermore, the effectors generated with or without IL-6 supplementation had a similar phenotype as determined by the expression of CD44, CD127, and Bcl-2 (Supplemental Figure 1).

### 3.3. IL-6 Is Dispensable for the Generation of CD8^+^ Tm Cells following Vaccination with DCs

**Figure 3:**
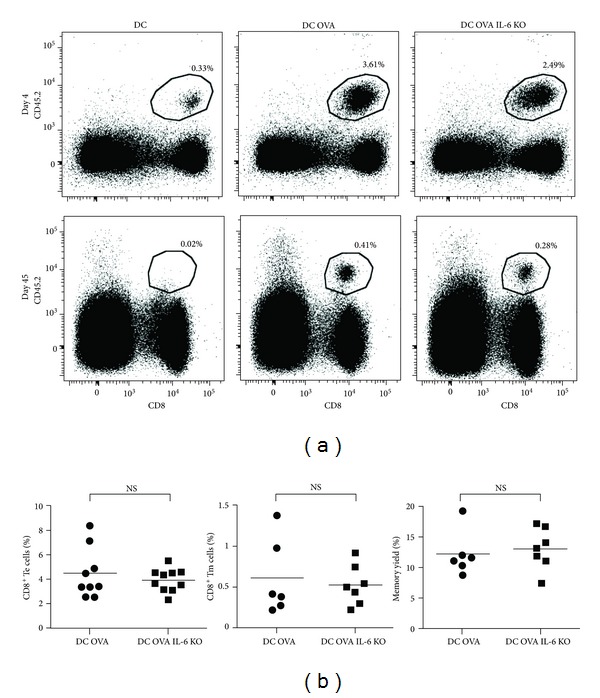
Normal generation of CD8^+^ effector and memory T cells following vaccination with IL-6-deficient DCs. (a) Vaccination with WT or IL-6 KO DCs generates OVA-specific CD8^+^ Te cells and Tm cells. 10^6^ female OT-I T cells (CD8^+^CD45.2^+^) were adoptively transferred into congenic B6.SJL female mice (CD45.1^+^) followed by immunization two days later with 0.5 × 10^6^ WT or IL-6 KO DCs, matured with LPS and loaded or not with OVA peptide. Te and Tm cells were identified as CD8^+^CD45.2^+^ by flow cytometry. The percentage of Te and Tm cells generated are indicated on each dot plot. (b) Quantification of CD8^+^ T cell response. Percentage of CD8^+^ Te (day 4, left panel) and Tm (day 45, middle panel) cells in one lymph node is shown. The yield of Tm cell formation was calculated as the percentage of Te cells that develop into Tm cells (right panel). The results are from two independent experiments with at least three mice per group. NS, non-significant.

Since it was possible that the amount administered and the route of injection did not lead to a sufficient IL-6 signals in naïve OT-I T cells, we tested whether IL-6 production by DCs was necessary for the generation of long-lived CD8^+^ Tm cells. To do so, we generated DCs from the bone marrow of IL-6-deficient mice. Before using these IL-6-deficient DCs in our vaccination protocol, we confirmed that they had a similar phenotype than wild-type DCs following LPS maturation (Supplemental Figure 2). Furthermore, IL-6-deficient DCs were equally loaded with the OVA peptide as WT DCs (Supplemental Figure 2). We then compared the OVA-specific CD8^+^ T-cell response following vaccination with IL-6-deficient or -sufficent DCs. As shown in Figure 3, a similar frequency and number (not shown) of CD8^+^ Te and Tm cells were generated following vaccination with WT or IL-6 KO DCs. Furthermore, the yield of CD8^+^ Tm cells (% of Te cells that developed into Tm cells) was similar in both groups (Figure 3(b)). These results show that IL-6 production by APCs at the priming of naïve CD8^+^ T cells is not necessary for the generation of CD8^+^ Te and Tm cells. Several reports have shown that IL-6 can enhance CD8^+^ T-cell proliferation *in vitro* [14, 20–24] and *in vivo* [14]. However, the use of IL-6-deficient DCs did not reduce the number of CD8^+^ Te cells generated. Thus, it is possible that the basal level of IL-6 present in the host is sufficient for optimal T-cell proliferation or that IL-6 production by DCs is not necessary for maximal proliferation of CD8^+^ T cells. Moreover, IL-6 production by CD40-B cells stimulated via the BCR and TLR7 was reported to be necessary for the maximal expansion of Ag-specific CD8^+^ T cells following vaccination [14]. Thus, our results with IL-6 KO DCs suggest that different APC types might produce different cytokines to promote the full expansion of CD8^+^ T cells. However, in our hands supplementation of IL-6 during CD40-B-cell vaccination did not increase T-cell expansion (Figure 2). This might be explained by the use of different stimuli (BCR + TLR7 ligand versus LPS) to mature the CD40B cells that may lead to production of different cytokines.

### 3.4. Vaccination with IL-6-Deficient DCs Generates Functional CD8^+^ Te and Tm Cells

**Figure 4:**
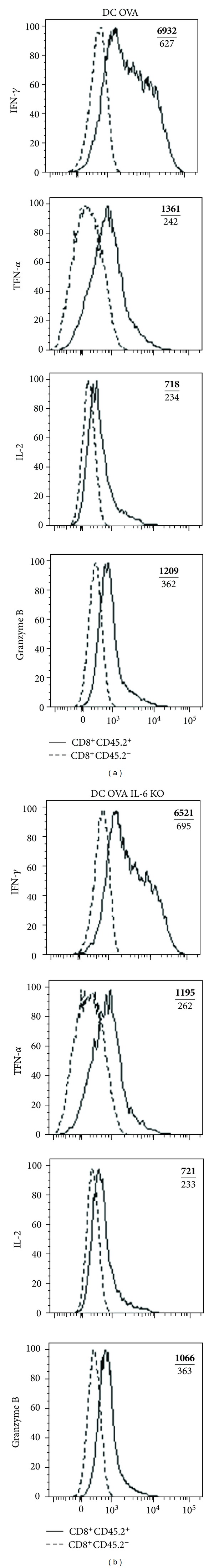
Generation of functional OVA-specific CD8^+^ effector T cells following immunization with IL-6-deficient DCs.Mice were immunized as in Figure 3 and effector molecules production was analyzed following a short *in vitro* stimulation with the OVA peptide. The overlays show production of the different effector molecules by OVA-specific Te cells (CD8^+^CD45.2^+^) compared to endogenous T cells (CD8^+^CD45.2^−^) at day 4 post-immunization with WT (left) or IL-6 KO (right) DCs. The MFI of effector molecule expression by OVA-specific CD8^+^ effectors (upper bold number) and endogenous CD8^+^ T cells (lower number) are indicated on each overlay.

**Figure 5:**
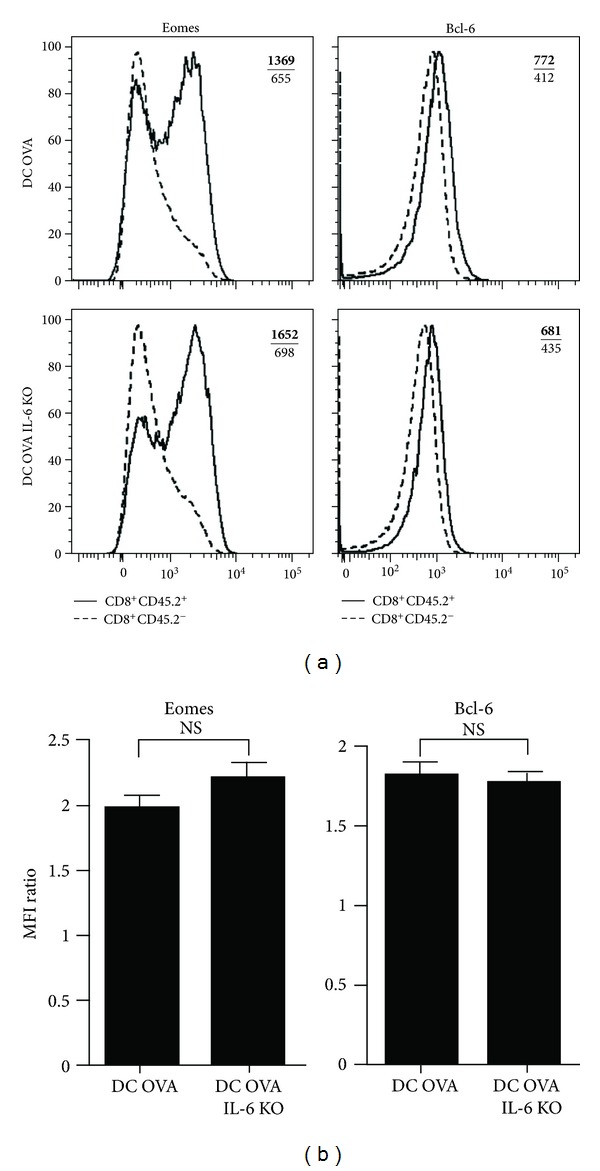
WT or IL-6 KO DC immunization generates effectors expressing similar levels of the transcription factors Eomes and Bcl-6. (a) The representative overlay histogram shows expression of Eomes and Bcl-6 by OVA-specific Te cells (CD8^+^CD45.2^+^) and endogenous T cells (CD8^+^CD45.2^−^). The MFI of Bcl-6 or Eomes expression by OVA-specific CD8^+^ effectors (upper bold number) and endogenous CD8^+^ T cells (lower number) are indicated on each overlay. Mice were immunized as in Figure 3. (b) Quantification of the level of expression of Eomes and Bcl-6. The bar charts show the MFI of expression for Eomes or Bcl-6 by OVA-specific CD8^+^ Te cells normalized to the MFI of endogenous CD8^+^ T cells. Results are presented as mean ± SEM. At least two mice per group.

 Since IL-6 was shown to influence cytotoxic T-cell differentiation [25], we have carefully evaluated the phenotype and functions of the OVA-specific CD8^+^ Te and Tm cells generated following vaccination with WT or IL-6 KO DCs. As shown in Figure 4, both types of effectors produce similar amounts of IFN-*γ*, TNF-*α*, IL-2, and granzyme B indicating that IL-6 signals from APCs at priming are not necessary for the acquisition of effector functions. Moreover, the OVA-specific CD8^+^ Te cells obtained with WT and IL-6 KO DCs express similar levels of CD44, CD127, 1B11, CD62L, CXCR3, and KLRG1 (Supplemental Figure 3). Furthermore, the OVA-specific CD8^+^ Te cells obtained with WT or IL-6 KO DCs have both undergone the proper differentiation program since they express similar level of Eomes and Bcl-6 (Figure 5), two key transcription factors controlling the differentiation of CD8^+^ Tm cells [35–40]. It is interesting to note that Bcl-6 expression is induced normally in CD8^+^ Te cells that have encountered the Ag on IL-6 KO DCs since IL-6 signals have been shown to influence the differentiation of follicular helper CD4^+^ T cells by modulating the expression level of Bcl-6 [41–43]. This suggests that the regulation of Bcl-6 expression is different in CD4^+^ versus CD8^+^ T cells or the endogenous source of IL-6 is sufficient to promote Bcl-6 expression in CD8^+^ Te cells. The proper differentiation of effectors following vaccination with IL-6 KO DCs contrasts with the results obtained by others where IL-6 induction by adjuvant was critical for cytotoxic T-cell differentiation [25]. One possible explanation is that vaccination with fully matured DCs bypassed the needs for IL-6. Altogether our results suggest that IL-6 production by the DCs involved in the priming of naïve CD8^+^ T cells is dispensable for the proper differentiation of CD8^+^ Te cells.

**Figure 6:**
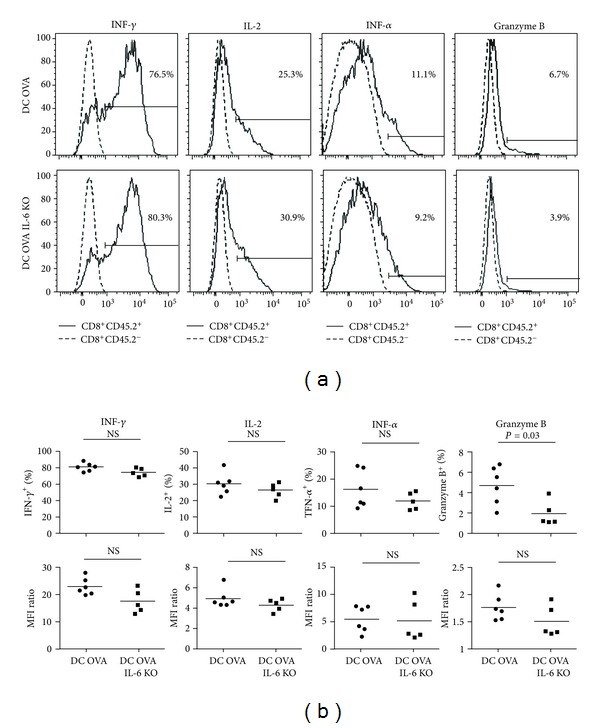
Generation of functional OVA-specific CD8^+^ memory T cells following immunization with IL-6-deficient DCs. (a) Functionality of OVA-specific CD8^+^ Tm cells at day 60 post-immunization. The overlays show production of the different effector molecules by OVA-specific T cells (CD8^+^CD45.2^+^) compared to endogenous T cells (CD8^+^CD45.2^−^) following immunization with WT (top) or IL-6 KO (bottom) DCs. The percentage of cells producing the different effector molecules is indicated on each histogram. (b) Quantification of cytokine and granzyme B production by OVA-specific CD8^+^ Tm cells. The percentage of cytokines and granzyme B producing OVA-specific Tm cells (top) and the amount produced (bottom) are shown at day 60 post-immunization. The MFI of cytokine and granzyme B production by CD8^+^ Tm cells was normalized to the MFI of the recipient CD8^+^ T cells (MFI ratio). The results are from two independent experiments.

Although CD8^+^ Tm cells were generated following vaccination with IL-6-deficient DCs, it was important to investigate if the Tm cells generated were fully functional. As shown in Figure 6, OVA-specific CD8^+^ Tm cells obtained with both WT and IL-6 KO DCs were similarly functional. They both produced similar amounts of IFN-*γ*, IL-2, TNF-*α* and granzyme B (Figure 6). These results show that IL-6 production by APCs during priming of naïve CD8^+^ T cells is also dispensable for the generation of fully functional CD8^+^ Tm cells. 

Our results show that IL-6 production by DCs is dispensable for the generation of fully functional CD8^+^ Tm cells. Furthermore, they also suggest that the lack of production of IL-6 by CD40-B cells is probably not the explanation for their inability to induce the formation of CD8^+^ Tm cells. Further studies are required to understand why CD40-B-cell vaccination does not promote the generation of CD8^+^ Tm cells. Possible explanations include differences in the site of priming, the level of costimulation, the interaction time with T cells, and the production of other soluble mediators such as IL-12 or type I IFNs. The ability of IL-6-deficient DCs to promote the generation of functional CD8^+^ Tm cells indicates that other soluble factors (IL-12 and IL-23) produced by DCs are sufficient to induce the generation of CD8^+^ Tm cells. Indeed, it was shown by others that vaccination with IL-12 and IL-23 deficient DCs abrogated CD8^+^ Tm-cell development [44]. It is also possible that IL-6 plays a role during CD8^+^ Tm-cell differentiation but that it does not have to be produced by the APCs involved in the T cell priming.

In conclusion, we show that the inability of CD40-B-cell vaccination to induce the formation of CD8^+^ Tm cells is not due to their reduced production of IL-6. Similarly, vaccination with IL-6-deficient DCs did not impede their ability to promote the formation of functional CD8^+^ Tm cells. Thus, IL-6 production by the APCs involved in the priming of naïve CD8^+^ T cells is dispensable for the formation of CD8^+^ Tm cells. Furthermore, our results also highlight the various role of IL-6 in different immunization protocol. Vaccination with DC does not rely on IL-6 for the full expansion and differentiation of CD8^+^ Te cells while IL-6 is necessary when adjuvant is used.

## Supplementary Material

Supplementary Figure S1: Shows the similar phenotype of CD8^*+*^ effectors generated after CD40-B cell immunization with or without IL-6 supplementation.
Supplementary Figure S2: Shows that wild-type and IL-6-deficient DCs have the same phenotypic characteristics has shown by their similar expression of CD11c, MHC class I and II molecules, CD86, CD80 and Kb-OVA.
Supplementary Figure S3: shows that CD8^*+*^ effector T cells obtained following immunization with wild-type or IL-6-deficient DCs express similar level of CD44, CD127, 1B11, CD62L, CXCR3 and KLRG1.

## Abbreviations Used in This Paper

Ag: Antigen
APC: Antigen-presenting cell
CD40-B cell: CD40-activated B cell
DC: Dendritic cell
IFN: Interferon
IL: Interleukin
KO: Knock-out
MPEC: Memory precursor effector cell
NS: Non-significant
OVA: Ovalbumin
R: Receptor
SEM: Standard error of the mean
SLEC: Short lived effector cell
Te cell: Effector T cell
Tm cell: Memory T cell
TNF: Tumor necrosis factor
WT: Wild-type.
