# Supplementary figures and images for: IL-6 Production by Dendritic Cells Is Dispensable for CD8+ Memory T-Cell Generation
Source: Biomed Res Int. 2012 Dec 30;2013:126189. doi: 10.1155/2013/126189 (PMC3591162; doi:10.1155/2013/126189)

## Slide 1
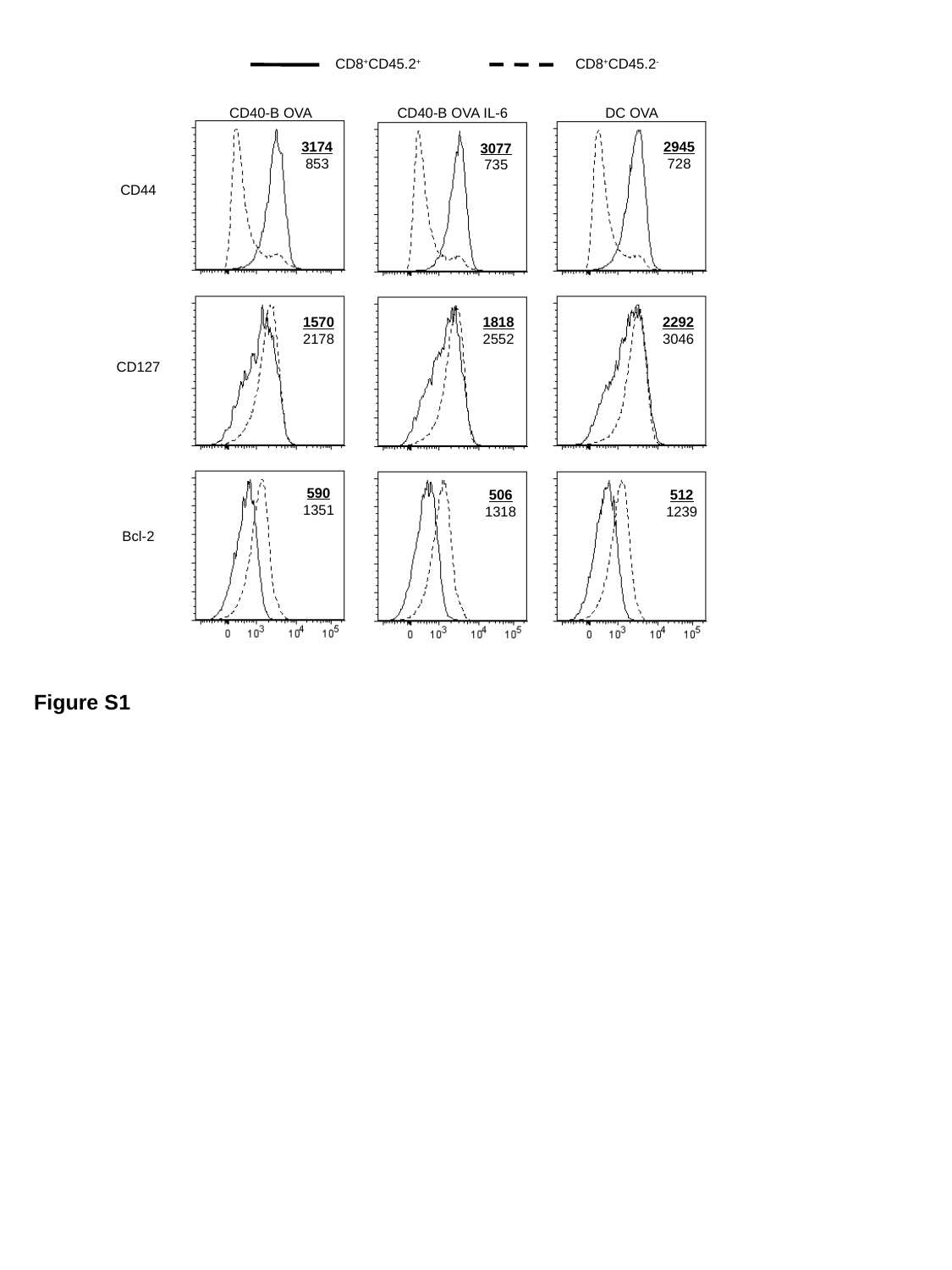

CD8+CD45.2+
CD8+CD45.2-
CD40-B OVA
CD40-B OVA IL-6
DC OVA
3174
853
2945
728
3077
735
CD44
1570
2178
1818
2552
2292
3046
CD127
590
1351
506
1318
512
1239
Bcl-2
Figure S1

Supplement: Supplementary file 1 — Supplementary Figure S1: Shows the similar phenotype of CD8+ effectors generated after CD40-B cell immunization with or without IL-6 supplementation. Supplementary Figure S2: Shows that wild-type and IL-6-deficient DCs have the same phenotypic characteristics has shown by their similar expression of CD11c, MHC class I and II molecules, CD86, CD80 and Kb-OVA. Supplementary Figure S3: shows that CD8+ effector T cells obtained following immunization with wild-type or IL-6-deficient DCs express similar level of CD44, CD127, 1B11, CD62L, CXCR3 and KLRG1. [file 126189.f1.ppt]

## Slide 1
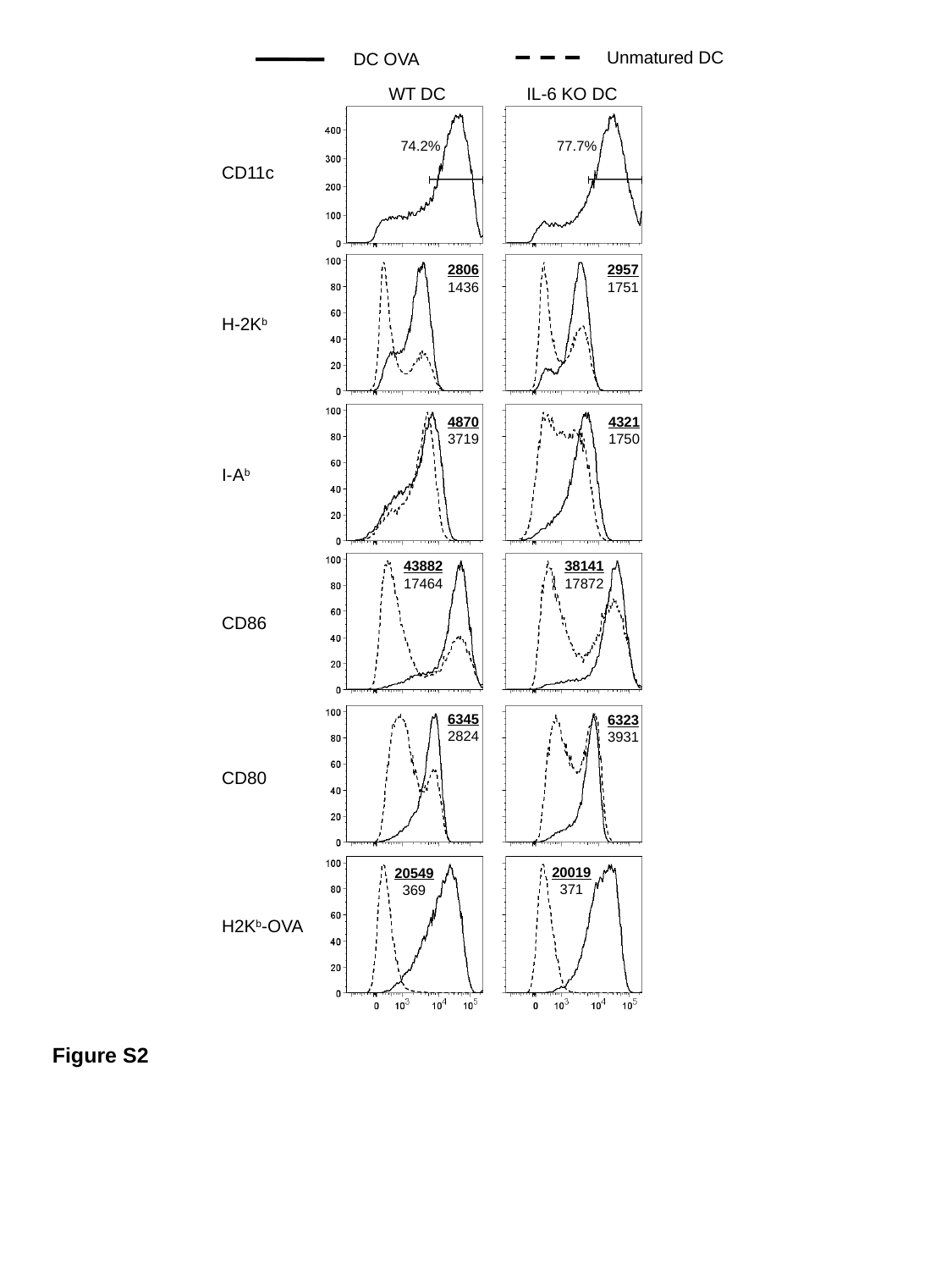

Unmatured DC
DC OVA
WT DC
IL-6 KO DC
74.2%
77.7%
CD11c
2806
1436
2957
1751
H-2Kb
4870
3719
4321
1750
I-Ab
43882
17464
38141
17872
CD86
6345
2824
6323
3931
CD80
20019
371
20549
369
H2Kb-OVA
Figure S2

Supplement: Supplementary file 2 [file 126189.f2.ppt]

## Slide 1
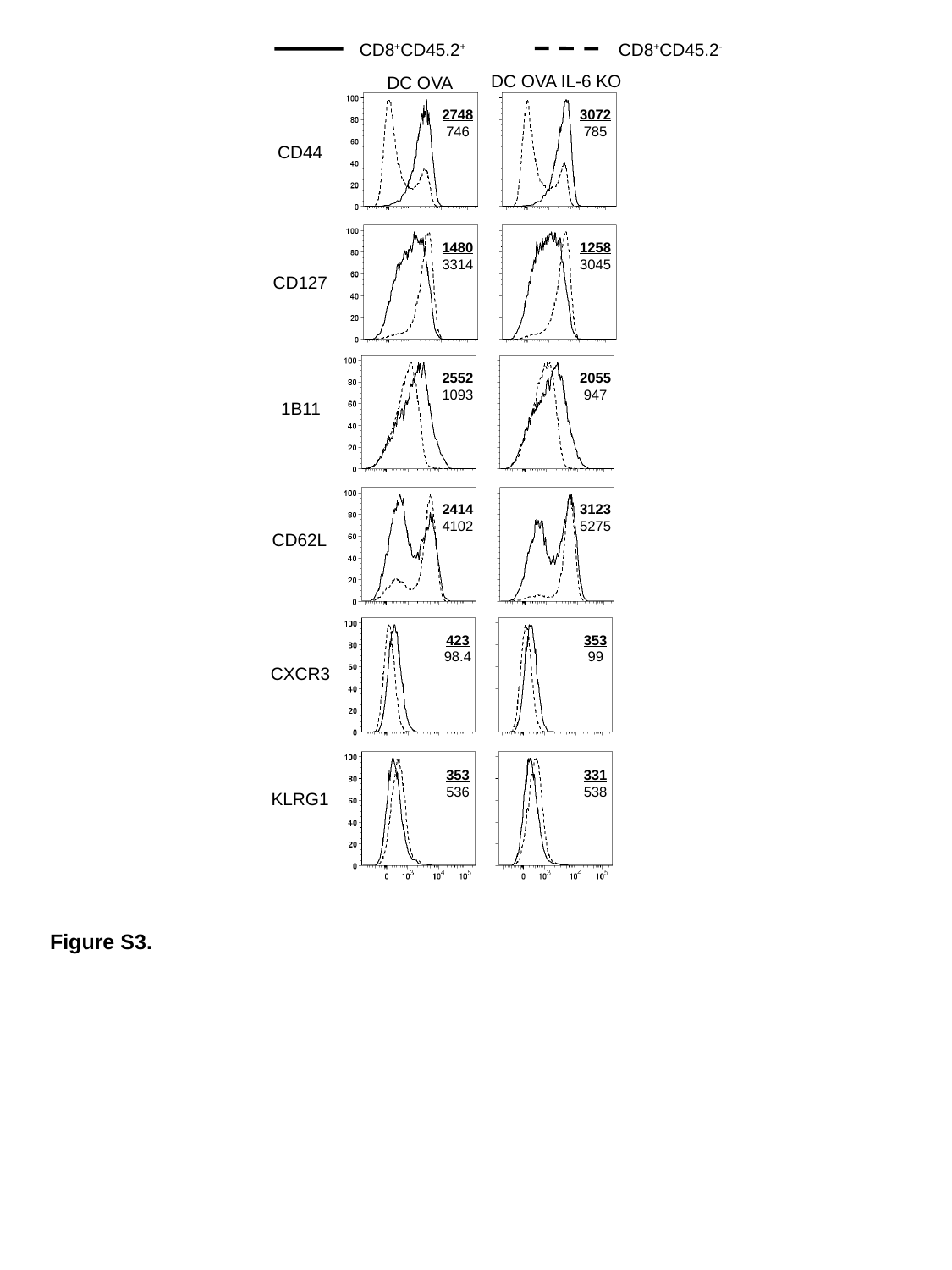

CD8+CD45.2+
CD8+CD45.2-
DC OVA IL-6 KO
DC OVA
2748
746
3072
785
CD44
1480
3314
1258
3045
CD127
2552
1093
2055
947
1B11
2414
4102
3123
5275
CD62L
423
98.4
353
99
CXCR3
353
536
331
538
KLRG1
Figure S3.

Supplement: Supplementary file 3 [file 126189.f3.ppt]
